# Genome sequence of the *Leisingera aquimarina* type strain (DSM 24565^T^), a member of the marine *Roseobacter* clade rich in extrachromosomal elements

**DOI:** 10.4056/sigs.3858183

**Published:** 2013-07-30

**Authors:** Thomas Riedel, Hazuki Teshima, Jörn Petersen, Anne Fiebig, Karen Davenport, Hajnalka Daligault, Tracy Erkkila, Wei Gu, Christine Munk, Yan Xu, Amy Chen, Amrita Pati, Natalia Ivanova, Lynne A. Goodwin, Patrick Chain, John C. Detter, Manfred Rohde, Sabine Gronow, Nikos C. Kyrpides, Tanja Woyke, Markus Göker, Thorsten Brinkhoff, Hans-Peter Klenk

**Affiliations:** 1 HZI – Helmholtz Centre for Infection Research, Braunschweig, Germany; 2 Los Alamos National Laboratory, Bioscience Division, Los Alamos, New Mexico, USA; 3 Leibniz Institute DSMZ - German Collection of Microorganisms and Cell Cultures, Braunschweig, Germany; 4 Biological Data Management and Technology Center, Lawrence Berkeley National Laboratory, Berkeley, California, USA; 5 DOE Joint Genome Institute, Walnut Creek, California, USA; 6 Institute for Chemistry and Biology of the Marine Environment (ICBM), Oldenburg, Germany

**Keywords:** marine, biofilm, ovoid-shaped, halotolerant, heterotrophic, quorum sensing, plasmid, thiosulfate oxidation, carbon monoxide utilization, *Rhodobacteraceae*, *Alphaproteobacteria*

## Abstract

*Leisingera aquimarina* Vandecandelaere *et al*. 2008 is a member of the genomically well characterized *Roseobacter* clade within the family *Rhodobacteraceae*. Representatives of the marine *Roseobacter* clade are metabolically versatile and involved in carbon fixation and biogeochemical processes. They form a physiologically heterogeneous group, found predominantly in coastal or polar waters, especially in symbiosis with algae, in microbial mats, in sediments or associated with invertebrates. Here we describe the features of *L. aquimarina* DSM 24565^T^ together with the permanent-draft genome sequence and annotation. The 5,344,253 bp long genome consists of one chromosome and an unusually high number of seven extrachromosomal elements and contains 5,129 protein-coding and 89 RNA genes. It was sequenced as part of the DOE Joint Genome Institute Community Sequencing Program 2010 and of the activities of the Transregional Collaborative Research Centre 51 funded by the German Research Foundation (DFG).

## Introduction

Strain R-26159^T^ (= DSM 24565^T^ = LMG 24366^T^ = CCUG 55860^T^) is the type strain of the species *Leisingera aquimarina* [1], one of the three species currently with a validly published name in the genus *Leisingera*; the other ones are the type species *L. methylohalidivorans* [1,2] and *L. nanhaiensis* [3]. The genus *Leisingera* is a member of the widespread *Roseobacter* clade, present in various marine habitats [4]. Strain R-26159^T^ was isolated from a marine electroactive biofilm grown on a stainless-steel cathode, which was exposed to natural seawater at the ISMAR-CNR Marine Station within the harbor of Genova (Italy) [1]. The genus *Leisingera* was named after Thomas Leisinger for his work on the bacterial methyl halide metabolism [2]; the species epithet *aquimarina* refers to the Neolatin adjective *marinus*, from the sea, from seawater. PubMed records do not currently indicate any follow-up research with strain R-26159^T^ after the initial description of *L. aquimarina* [1].

Here we present a summary classification and a set of features for *L. aquimarina* DSM 24565^T^, together with the description of the genomic sequencing and annotation.

## Classification and features of the organism

### 16S rRNA analysis

A representative genomic 16S rRNA gene sequence of *L. aquimarina* DSM 24565^T^ was compared using NCBI BLAST [5,6] under default settings (e.g., considering only the high-scoring segment pairs (HSPs) from the best 250 hits) with the most recent release of the Greengenes database [7] and the relative frequencies of taxa and keywords (reduced to their stem [8]) were determined, weighted by BLAST scores. The most frequently occurring genera were *Phaeobacter* (31.4%), *Ruegeria* (25.9%), *Silicibacter* (16.1%), *Roseobacter* (14.4%) and *Nautella* (3.9%) (127 hits in total). Regarding the four hits to sequences from other members of the genus, the average identity within HSPs was 99.4%, whereas the average coverage by HSPs was 99.3%. Among all other species, the one yielding the highest score was *Leisingera methylohalidivorans* (NR_025637), which corresponded to an identity of 99.2% and an HSP coverage of 100.0%. (Note that the Greengenes database uses the INSDC (= EMBL/NCBI/DDBJ) annotation, which is not an authoritative source for nomenclature or classification.) The highest-scoring environmental sequence was FJ202534 (Greengenes short name 'and White Plague Disease-Induced Changes Caribbean Coral *Montastraea faveolata* kept aquarium 23 days clone SGUS1024'), which showed an identity of 97.8% and an HSP coverage of 100.0%. The most frequently occurring keywords within the labels of all environmental samples which yielded hits were 'coral' (4.7%), 'caribbean' (3.8%), 'faveolata' (3.5%), 'chang' (3.4%) and 'white' (3.3%) (117 hits in total). Environmental samples which yielded hits of a higher score than the highest scoring species were not found, which might indicate that the species is rarely found in the environment.

**Figure 1:**
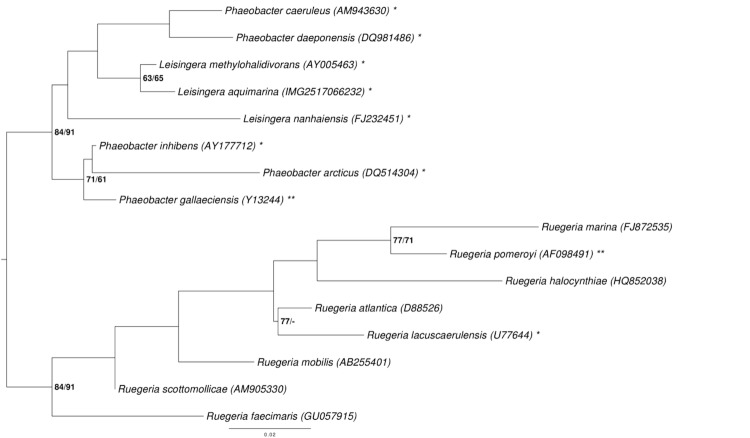
Phylogenetic tree highlighting the position of *L. aquimarina* relative to the type strains of the other species within the genus *Leisingera* and the neighboring genera *Phaeobacter* and *Ruegeria*. The tree was inferred from 1,383 aligned characters [9,10] of the 16S rRNA gene sequence under the maximum likelihood (ML) criterion [11]. Rooting was done initially using the midpoint method [12] and then checked for its agreement with the current classification (Table 1). The branches are scaled in terms of the expected number of substitutions per site. Numbers adjacent to the branches are support values from 1,000 ML bootstrap replicates [13] (left) and from 1,000 maximum-parsimony bootstrap replicates [14] (right) if larger than 60%. Lineages with type strain genome sequencing projects registered in GOLD [15] are labeled with one asterisk, those also listed as 'Complete and Published' with two asterisks [16,17]. The genomes of *P. caeruleus* [58] and *P. arcticus* [59] are reported in this issue.

Figure 1 shows the phylogenetic neighborhood of *L. aquimarina* in a 16S rRNA gene based tree. The sequences of the four identical 16S rRNA gene copies in the genome do not differ from the previously published 16S rRNA gene sequence AM900415.

**Table 1:** Classification and general features of *L. aquimarina* DSM 24565^T^ according to the MIGS recommendations [18].

MIGS ID	Property	Term	Evidence code
		Domain *Bacteria*	TAS [19]
		Phylum *Proteobacteria*	TAS [20]
		Class *Alphaproteobacteria*	TAS [21,22]
	Classification	Order *Rhodobacterales*	TAS [22,23]
		Family *Rhodobacteraceae*	TAS [22,24]
		Genus *Leisingera*	TAS [25,26]
		Species *Leisingera aquimarina*	TAS [1]
MIGS-7	Subspecific genetic lineage (strain)	R-26159^T^	TAS [1]
MIGS-12	Reference for biomaterial	Vandecandelaere *et al.* 2008	TAS [1]
	Current classification		
	Gram stain	Negative	TAS [1]
	Cell shape	Ovoid-shaped	TAS [1]
	Motility	Motile	TAS [1]
	Sporulation	Not reported	
	Temperature range	Mesophile (4 – 37°C)	TAS [1]
	Optimum temperature	20°C	NAS
	Salinity	Halophile, 1-7% NaCl (w/v)	TAS [1]
MIGS-22	Relationship to oxygen	aerobic	TAS [1]
	Carbon source	Yeast extract, peptone, betaine	TAS [1]
MIGS-6	Habitat	Seawater, biofilm	TAS [1]
MIGS-6.2	pH	6.5 – 8.0	TAS [1]
MIGS-15	Biotic relationship	Free living	TAS [1]
	Biosafety level	1	TAS [27]
MIGS-23.1	Isolation	Marine biofilm on stainless steel cathode	TAS [1]
MIGS-4	Geographic location	ISMAR-CNR Marine Station, Genoa harbor, Italy	TAS [1]
MIGS-4.1	Latitude	+44.41	TAS [1]
MIGS-4.2	Longitude	+8.92	TAS [1]
MIGS-4.3	Depth	Not reported	

Evidence codes - TAS: Traceable Author Statement (i.e., a direct report exists in the literature); NAS: Non-traceable Author Statement (i.e., not directly observed for the living, isolated sample, but based on a generally accepted property for the species, or anecdotal evidence). Evidence codes are from the Gene Ontology project [28].

### Morphology and physiology

**Figure 2:**
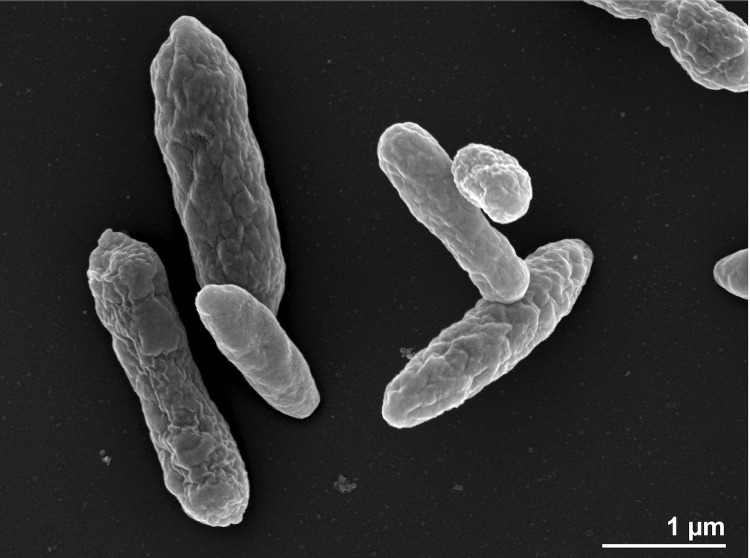
Scanning electron micrograph of *L. aquimarina* DSM 24565^T^.

Cells of strain R-26159^T^ are Gram-negative, ovoid (1 × 1.4 µm) and contain a single polar flagellum (not visible in Figure 2), which is used for motility. Poly-β-hydroxybutyrate is present in inclusion bodies. Colonies are dark beige-pink in color, round and 1–2 mm in diameter after 3 days incubation on marine agar (MA). Growth occurs after 2 days incubation at 20 °C on MA, but not on Reasoner’ 2A agar (R2A), Nutrient agar (NA), Trypticase-Soy agar (TSA) or Peptone-Yeast Extract-Glucose agar (PYG). The temperature range for growth is 4–37°C whereas no growth occurs at 40°C or higher. The salinity range for growth is 1–7% NaCl. The pH range for growth is 5.5–9.0 with an optimum between 6.5–8. Growth occurs on betaine (1 mM) as a sole carbon source, but not on L-methionine (10 mM). Cells are catalase- and oxidase-positive. Degradation of gelatin is weakly positive but cells do not degrade tyrosine, DNA, starch, casein, chitin, aesculin or Tween 80. The strain shows leucine arylamidase activity; weak alkaline phosphatase, esterase lipase (C8) and naphthol-AS-BI phosphohydrolase activities. No activity is detected for esterase (C4), valine arylamidase, acid phosphatase, *α*-galactosidase, *β*-glucuronidase, *α*-glucosidase, *β*-glucosidase, *N*-acetyl-*β*-glucosaminidase, *α*-mannosidase, lipase (C14), cystine arylamidase, trypsin, *α*-chymotrypsin, arginine dihydrolase, urease or *α*-fucosidase. Nitrate is not reduced to nitrite or nitrogen. Indole is not produced and glucose is not fermented. Cells do not assimilate D-glucose, L-arabinose, D-mannose, D-mannitol, *N*-acetylglucosamine, maltose, potassium gluconate, capric acid, adipic acid, malate, citrate or phenylacetic acid. Cells are susceptible to cefoxitin (30 mg), erythromycin (15 mg), tetracycline (30 mg) and streptomycin (25 mg), but resistant to vancomycin (30 mg), trimethoprim (1.25 mg), clindamycin (2 mg) and gentamicin (30 mg) (all data from [1]).

The utilization of carbon compounds by *L. aquimarina* DSM 24565^T^ grown at 20°C was also determined for this study using Generation-III microplates in an OmniLog phenotyping device (BIOLOG Inc., Hayward, CA, USA). The microplates were inoculated at 28°C with a cell suspension at a cell density of 95-96% turbidity and dye IF-A. Further additives were vitamin, micronutrient and sea-salt solutions. The exported measurement data were further analyzed with the opm package for R [29,60], using its functionality for statistically estimating parameters from the respiration curves and translating them into negative, ambiguous, and positive reactions. The strain was studied in two independent biological replicates, and reactions with a different behavior between the two repetitions were regarded as ambiguous. At 28°C the strain reacted poorly, with positive reactions only for 1% NaCl, 4% NaCl and lithium chloride. This is in accordance with the comparatively low median of the temperature range of the strain [1].

### Chemotaxonomy

The principal cellular fatty acids of strain R-26159^T^ are mono-unsaturated straight chain acids: C_18:1 ω7c_ (71.6%), C_14:1_ *_iso_*_E_ (11.6%), C_14:1 2-OH_ (4.2%), C_16:0 2-OH_ (4.2%), C_16:0_ (3.5%), an unknown fatty acid of equivalent chain-length 11.799 (2.7%), C_12:0_ 3-OH (2.1%) as well as C_10:0 3-OH_ (2.0%) [26]. Remaining fatty acids were detected in very small fractions only (<1.0%) [1]. The same predominant fatty acids were also found in other members of the *Phaeobacter*-*Leisingera* cluster [2,26,30].

## Genome sequencing and annotation

### Genome project history

This organism was selected for sequencing on the basis of the DOE Joint Genome Institute **C**ommunity **S**equencing **P**rogram 2010, CSP 441: “Whole genome type strain sequences of the genera *Phaeobacter* and *Leisingera* – a monophyletic group of highly physiologically diverse organisms”. The genome project is deposited in the GenomesOnLine Database [15] and the complete genome sequence was submitted to GenBank. Sequencing, finishing and annotation were performed by the DOE Joint Genome Institute (JGI). A summary of the project information is shown in Table 2.

**Table 2:** Genome sequencing project information

MIGS ID	Property	Term
MIGS-31	Finishing quality	Improved high quality draft
MIGS-28	Libraries used	Two genomic libraries: Illumina standard (short PE), Illumina CLIP (long PE)
MIGS-29	Sequencing platforms	Illumina HiSeq 2000, PacBio
MIGS-31.2	Sequencing coverage	699 × Illumina; unknown × PacBio
MIGS-30	Assemblers	Allpath version 39750, Velvet 1.1.05, phrap version SPS - 4.24
MIGS-32	Gene calling method	Prodigal 1.4, GenePRIMP
	INSDC ID	*pending*
	GenBank Date of Release	*pending*
	GOLD ID	Gi10856
	NCBI project ID	81653
	Database: IMG	2521172617
MIGS-13	Source material identifier	DSM 24565^T^
	Project relevance	Tree of Life, carbon cycle, sulfur cycle, environmental

### Growth conditions and DNA isolation

A culture of *L. aquimarina* DSM 24565^T^ was grown in the DSMZ medium 514 (BACTO Marine Broth) [31] at 20°C. Genomic DNA was isolated from 0.5-1 g of cell paste using Jetflex Genomic DNA Purification Kit (GENOMED 600100) following the standard protocol provided by the manufacturer but modified by the use of additional 20 µl proteinase K and 40 minute incubation. DNA is available through the DNA Bank Network [32].

### Genome sequencing and assembly

The draft genome was generated using Illumina data [33]. For this genome, we constructed and sequenced an Illumina short-insert paired-end library with an average insert size of 270 bp which generated 13,668,574 reads and an Illumina long-insert paired-end library with an average insert size of 8047.58 +/- 2682.23 bp which generated 11,512,166 reads totaling 3,777 Mbp of Illumina data (Feng Chen, unpublished). All general aspects of library construction and sequencing can be found at the JGI web site [34]. The initial draft assembly contained 64 contigs in 18 scaffold(s). The initial draft data was assembled with Allpaths [35] and the consensus was computationally shredded into 10 kbp overlapping fake reads (shreds). The Illumina draft data was also assembled with Velvet [36], and the consensus sequences were computationally shredded into 1.5 kbp overlapping fake reads (shreds). The Illumina draft data was assembled again with Velvet using the shreds from the first Velvet assembly to guide the next assembly. The consensus from the second Velvet assembly was shredded into 1.5 kbp overlapping fake reads. The fake reads from the Allpaths assembly and both Velvet assemblies and a subset of the Illumina CLIP paired-end reads were assembled using parallel phrap (High Performance Software, LLC) [37]. Possible mis-assemblies were corrected with manual editing in Consed [34,36,37]. Gap closure was accomplished using repeat resolution software (Wei Gu, unpublished), and sequencing of bridging PCR fragments with PacBio (Cliff Han, unpublished) technologies. A total of 57 PCR PacBio consensus sequences were completed to close gaps and to raise the quality of the final sequence. The final assembly is based on 3,777 Mbp of Illumina draft data, which provides an average 699 × coverage of the genome.

### Genome annotation

Genes were identified using Prodigal [38] as part of the JGI genome annotation pipeline [39], followed by a round of manual curation using the JGI GenePRIMP pipeline [40]. The predicted CDSs were translated and used to search the National Center for Biotechnology Information (NCBI) nonredundant database, UniProt, TIGR-Fam, Pfam, PRIAM, KEGG, COG, and InterPro databases. Additional gene prediction analysis and functional annotation was performed within the Integrated Microbial Genomes - Expert Review (IMG-ER) platform [41].

## Genome properties

**Figure 3:**
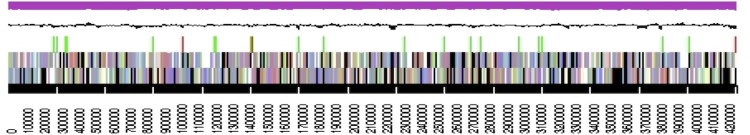
Graphical map of the chromosome. From bottom to the top: Genes on forward strand (colored by COG categories), Genes on reverse strand (colored by COG categories), RNA genes (tRNAs green, rRNAs red, other RNAs black), GC content (black), GC skew (purple/olive).

The genome statistics are provided in Table 3 and Figure 3. The genome consists of a 4.25 Mbp chromosome and seven extrachromosomal elements of 6.2 to 248.9 kbp length with a G+C content of 61.4%. Of the 5,218 genes predicted, 5,129 were protein-coding genes, and 89 RNAs. The majority of the protein-coding genes (80.4%) were assigned a putative function while the remaining ones were annotated as hypothetical proteins. The distribution of genes into COGs functional categories is presented in Table 4.

**Table 3:** Genome Statistics

**Attribute**	**Value**	**% of Total**
Genome size (bp)	5,344,253	100.00
DNA coding region (bp)	4,678,916	87.55
DNA G+C content (bp)	3,278,568	61.35
Number of scaffolds	8	
Extrachromosomal elements	7	
Total genes	5,218	100.00
RNA genes	89	1.71
rRNA operons	4	
tRNA genes	61	1.17
Protein-coding genes	5,129	98.29
Genes with function prediction (proteins)	4,196	80.41
Genes in paralog clusters	4,110	78.77
Genes assigned to COGs	3,955	75.80
Genes assigned Pfam domains	4,253	81.51
Genes with signal peptides	419	8.03
Genes with transmembrane helices	1,037	19.87
CRISPR repeats	0	

**Table 4:** Number of genes associated with the general COG functional categories

**Code**	**value**	**%age**	**Description**
J	175	4.0	Translation, ribosomal structure and biogenesis
A	1	0.0	RNA processing and modification
K	365	8.4	Transcription
L	296	6.8	Replication, recombination and repair
B	2	0.1	Chromatin structure and dynamics
D	43	1.0	Cell cycle control, cell division, chromosome partitioning
Y	0	0.0	Nuclear structure
V	53	1.2	Defense mechanisms
T	182	4.2	Signal transduction mechanisms
M	221	5.1	Cell wall/membrane/envelope biogenesis
N	55	1.3	Cell motility
Z	2	0.1	Cytoskeleton
W	0	0.0	Extracellular structures
U	75	1.7	Intracellular trafficking and secretion, and vesicular transport
O	157	3.6	Posttranslational modification, protein turnover, chaperones
C	273	6.3	Energy production and conversion
G	168	3.9	Carbohydrate transport and metabolism
E	550	12.6	Amino acid transport and metabolism
F	95	2.2	Nucleotide transport and metabolism
H	188	4.3	Coenzyme transport and metabolism
I	172	4.0	Lipid transport and metabolism
P	220	5.1	Inorganic ion transport and metabolism
Q	152	3.5	Secondary metabolites biosynthesis, transport and catabolism
R	513	11.8	General function prediction only
S	393	9.0	Function unknown
-	1,263	24.2	Not in COGs

### Insights into the genome

Genome sequencing of *Leisingera aquimarina* DSM 24565^T^ reveals the presence of seven plasmids with sizes between 6 kb and 249 kb (Table 5). The circular conformation of the chromosome and the two smallest extrachromosomal elements has been experimentally validated. The six larger plasmids contain characteristic replication modules [42] of the RepABC-, DnaA-like, RepA- and RepB-type comprising a replicase as well as the *parAB* partitioning operon [43]. The respective replicases that mediate the initiation of replication are designated according to the established plasmid classification scheme [44]. The different numbering of e.g. the replicases RepC-8, RepC-13 and RepC-14 from RepABC-type plasmids corresponds to specific plasmid compatibility groups that are required for a stable coexistence of the replicons within the same cell [45]. The cryptic 6 kb plasmid pAqui_G6 contains a solitary RepA-II type replicase without a partitioning module, but replicon maintenance in the daughter cells is probably ensured by its postsegregational killing system (PSK) consisting of a typical operon with two small genes encoding a stable toxin and an unstable antitoxin [46]. PSK systems are also located on pAqui_C182 and pAqui_F126 (Tab. 6).

**Table 5:** General genomic features of the chromosome and extrachromosomal replicons from *Leisingera aquimarina* strain DSM 24565^T^.

**Replicon**	**Scaffold**	**Replicase**	**Length** (bp)	**GC** (%)	**Topology**	**No. Genes^#^**
Chromosome	1	DnaA	4,250,010	61	circular	4245
pAqui_A249	2	RepC-14	248,908	59	linear*	238
pAqui_B243	3	RepC-13	242,809	61	linear*	231
pAqui_C182	4	RepC-8	182,150	63	linear*	159
pAqui_D148	5	RepB-I	148,175	63	linear*	121
pAqui_E140	6	RepA-I	140,244	62	linear*	109
pAqui_F126	7	DnaA-like I	125,793	62	circular	105
pAqui_G6	8	RepA-II	6,164	58	circular	10

^*^Circularity not experimentally validated; ^#^deduced from automatic annotation.

**Table 6:** Integrated Microbial Genome (IMG) locus tags of *L. aquimarina* DSM 24565^T^ genes for the initiation of replication, toxin/antitoxin modules and two representatives of type IV secretion systems (T4SS) that are required for conjugation. The locus tags are accentuated in blue^1,2,3^.

	**Replication Initiation**		**Plasmid Stability**		**Type IV Secretion**	
**Replicon**	Replicase	Locus Tag	Toxin	Antitoxin	VirB4	VirD4
Chromosome	DnaA	Aqui_0952	-	-	Aqui _3705	Aqui _3720^³^
pAqui_A249	RepC-14	Aqui_4671	-	-	Aqui _4685^²^	Aqui _4598^³^
pAqui_B243	RepC-13	Aqui_4931	-	-	-	-
pAqui_C182	RepC-8	Aqui_5105	Aqui_5145	Aqui_5144	-	-
pAqui_D148	RepB-I	Aqui_4343	-	-	-	-
pAqui_E140	RepA-I	Aqui_4076	-	-	-	-
pAqui_F126	DnaA-like I	Aqui_4459	Aqui_4464	Aqui_4465		
pAqui_G6	RepA-II¹	Aqui_5224	Aqui_5228	Aqui_5229	-	-

¹solitary replicase without partitioning module; ^²^*traC* gene of F factor conjugation system; ^3^presence of adjacent DNA relaxase VirD2.

The locus tags of all replicases, plasmid stability modules and the large *virB4* and *virD4* genes of type IV secretion systems are presented in Table 6. A characteristic T4SS comprising the relaxase VirD2 and the coupling protein VirD4 as well as the complete *virB* gene cluster for the transmembrane channel is located on the chromosome [47]. Its functional role is unclear, but very closely related T4SS are detected on plasmids of e.g. *Dinoroseobacter shibae* DSM 16493^T^ [61], *Leisingera nanhaiensis* DSM 24252^T^ and *Phaeobacter caeruleus* DSM 24564^T^ [48]. Furthermore, the largest plasmid pAqui_A249 contains the complete F factor conjugation transfer (*tra*) region with 20 genes (Aqui_4678 to Aqui_4697). It exhibits only weak homology with the typical type IV secretion system of the *Roseobacter* clade, which is represented by the chromosomal counterpart, but it resembles the F sex factor of *Escherichia coli* that is the paradigm for bacterial conjugation [49].

The 243 kb RepABC-13 type plasmid pAqui_B243 is predominated by seven ABC-transporters. Even more conspicuous is the presence of a couple of pentose phosphate pathway genes including an operon of genes of the Entner-Doudoroff pathway (Aqui_4914 to Aqui_4917; EC 1.1.1.49; EC 4.2.1.12; EC 4.1.2.14; EC 5.3.1.9) that is generally used in *Roseobacters* to convert D-fructose-6-phosphate to D-glyceraldehyde-3-phosphate [50]. The exclusive missing gene within this operon is the chromosome encoded 6-phosphogluconolactonase (Aqui_2983; EC 3.1.1.31). The presence of a glycolytic 6-phosphofructokinase (PFK; Aqui_4950; EC 2.7.1.11) is a genetic novelty in this group of marine bacteria, because the current opinion was that “*the typical pfk gene is absent from all sequenced Roseobacter clade genomes and glucose is hence probably catabolized via the Entner–Doudoroff pathway and not via classical glycolysis*” [51]. However, the putative functionality of the Embden-Meyerhoff-Parnas pathway (glycolysis) has to be validated e.g. via pulse-chase experiments with 13C labeled glucose [52]. Finally, the plasmid pAqui_B243 contains the phosphoenolpyruvate synthase (Aqui_4951; EC 2.7.1.11) that is required together with the chromosomal phosphoenolpyruvate carboxylase (Aqui_0364; EC 4.1.1.31) for prokaryotic CO_2_ fixation and the formation of oxaloacetate from pyruvate.

The 148 kb RepB-I type plasmid pAqui_D148 contains a complete rhamnose operon [47] and many genes that are required for polysaccharide biosynthesis. This extrachromosomal replicon also harbors two siderophore synthetase genes (Aqui_4320; Aqui_4321), two outer membrane receptors for Fe-transport (Aqui_4319; Aqui_4360) and genes of a putative ABC-type Fe^3+^ siderophore transport system (Aqui_4361 to Aqui_4364).

The 140 kb RepA-I type plasmid pAqui_E140 is largely predominated by glycosyltransferases, polysaccharide biosynthesis as well as cell-wall biogenesis genes, and it contains an operon for GDP-mannose metabolism (Aqui_5058 to Aqui_5055).

The 126 kb DnaA-like I replicon pAqui_F126 contains a large type VI secretion system (T6SS) with a size of about 30 kb. The role of this export system that has been first described in the context of bacterial pathogenesis, but recent findings indicate a more general physiological role in defense against eukaryotic cells and other bacteria in the environment [53]. Homologous T6S systems are present on the DnaA-like I plasmids of *Leisingera methylohalidivorans* DSM 14336^T^ (pMeth_A285) and *Phaeobacter caeruleus* DSM 24564^T^ (pCaer_C109) as well as the RepC-8 type plasmid of *Phaeobacter daeponensis* DSM23529^T^ (pDaep_A276).

Genome analysis of strain *L. aquimarina* DSM 24565^T^ revealed further the presence of genes encoding *LuxI* as well as *LuxR* homologues, which are involved in quorum sensing (QS), an already known feature of several members of the *Roseobacter* clade [54]. QS is a bacterial communication system used by many bacterial species to coordinate special behaviors based on bacterial population density [54]. Whereas two genes encode a N-acyl-L-homoserine lactone synthase (*LuxI*, Aqui_0074, Aqui_4264), some genes were identified to encode *LuxR* homologues (response and transcriptional regulators, e.g., Aqui_0075 and Aqui_3114).

Furthermore, several genes forming a putative operon are involved in the oxidation of (e.g., Aqui_3422 to Aqui_3426) indicating the oxidation of thiosulfate into sulfate to produce energy. Additionally genes for carbon monoxide utilization (Aqui_2391 and Aqui_2392, Aqui_2518, Aqui_2520, Aqui_3522, Aqui_5216 and Aqui_5217) were observed.

Interestingly, also a gene encoding a sensor of blue light using FAD (BLUF, Aqui_2375) was detected, indicating possible blue-light depending signal transduction.

As indicated by the 16S rRNA gene sequence analysis (Figure 1), the classification of some *Leisingera* and *Phaeobacter* species might need to be reconsidered. We conducted a preliminary phylogenomic analysis with GGDC [55-57] applied to the genome of *L. aquimarina* DSM 24565^T^ and the draft genomes of the type strains of the other *Leisingera* and *Phaeobacter* species. The results shown in Table 7 indicate that the DNA-DNA hybridization (DDH) similarities calculated *in silico* of *L. aquimarina* to *Phaeobacter caeruleus* and *P. daeponensis* species are higher than those to *L. nanhaiensis*, confirming the 16S rRNA gene sequence analysis. Thus a taxonomic revision of *L. aquimarina* might be warranted.

**Table 7:** DDH similarities between *L. aquimarina* DSM 24565^T^ and the other *Leisingera* and *Phaeobacter* species (with genome-sequenced type strains) ^†^

**Reference species**	**formula 1**	**formula 2**	**formula 3**
*L. nanhaiensis* (2512047090)	14.50±3.11	19.20±2.28	14.70±2.65
*L. methylohalidivorans* (2512564009)	52.40±3.47	32.40±2.46	47.00±3.03
*P. arcticus* (2516653081)	16.60±3.25	20.70±2.32	16.50±2.75
*P. caeruleus* (2512047087)	45.90±3.41	28.40±2.44	40.60±3.01
*P. daeponensis* (2516493020)	47.30±3.42	27.90±2.43	41.30±3.01
*P. gallaeciensis* (AOQA01000000)	17.90±3.31	21.50±2.34	17.60±2.80
*P. inhibens* (2516653078)	18.30±3.33	20.80±2.33	17.90±2.82

**^†^** DDH similarities were calculated *in silico* with the GGDC server version 2.0 [57]. The standard deviations indicate the inherent uncertainty in estimating DDH values from intergenomic distances based on models derived from empirical test data sets (which are always limited in size); see [56] for details. The distance formulas are explained in [56]. The numbers in parentheses are IMG object IDs (GenBank accession number in the case of *P. gallaeciensis*) identifying the underlying genome sequences.
